# Inquiry into the Number of Victims by Lightning, and into Certain Phenomena Observed in Men and Animals Struck by Lightning

**Published:** 1855-02

**Authors:** 

**Affiliations:** Physician in Chief to the Military Hospital of Roule


*Inquiry into the number of victims by Lightning, and into certain Phenomena observed in Men and Animals struck by Lightning.* By M. Boudin, Physician in Chief to the Military Hospital of Roule. (*Memoir read before the Academy of Sciences, sitting of the 23d of October.*)

(Translated from the French for the Medical Examiner.)

The dissertation which we have the honor to submit to the Academy has for its object to call attention to, 1st, the great number of deaths by lightning, and of accidents caused by it.

2dly. To certain phenomena observed in men and animals struck by lightning.

If means capable of protecting man, dwellings and vessels against lightning are too frequently neglected, it is because the extent of the evil is very generally unknown.

Besides, the medical history of lightning-stroke has been, hitherto, so little studied, that the signs by which death by it has been recognized are not to be found in any of the treatises on legal medicine. From this double claim, our discourse will not, perhaps, be devoid of a life-like interest.

"The number of persons destroyed by lightning is so limited," said M. Arago, "that the probability of death by such means may be considered as trifling. The journals of 1805 did not record a single death by lightning in France. In 1806, the death of but two children was mentioned; in 1807 two husbandmen only were struck; in 1808 but one waterman is mentioned as having been killed by this agent." Such was the statement of M. Arago.

According to M. Kœmtz, the fear of lightning arises from *early prejudice, inculcated by ignorant parents.*

As regards the opinions of these two learned men, if we interrogate facts, we shall find that, in the short period from 1835 to 1852, not less than thirteen hundred and eight persons were destroyed by lightning in France. We speak only of those who were *instantaneously* killed. As for our documents, we have taken them from the recording office of the Minister of Justice. The number of persons killed by lightning has increased, in 1835, to 111; in 1847 to 108 persons. It is evident, however, that the number who were *instantaneously* killed does not include all who were victims to this cause.

In 1797, Volney mentioned that, in the United States, in three months, 17 persons were killed by lightning and 84 seriously injured. From these data, we think that the number of persons struck by lightning should be estimated to be at least three times greater than that of those instantaneously killed. It should hence follow, that the mean of persons struck by lightning in France annually exceeds two hundred in number.

In consulting other official documents, we have found the annual mean number of individuals killed, *at once*, by lightning in other countries to be, in Belgium, 3; in Sweden, 9·64; in England 22.

We have constructed a geographical chart, making a division into departments, of the deaths caused by lightning. From this document it follows,

1st. That no department completely escapes from such accidents.

2d. That they are very unequally distributed in the different departments.

3d. That the maximum of deaths by lightning is found in those departments which combine to form the central table-land of France and a few other mountainous departments.

Thus, in the period we have examined, we find two deaths in Eure, three in Eure et Loir and Calvados, which number increases to 20 deaths in Cantal, 24 in Aveyron, 27 in Corsica, 38

in Saône-et-Loire, 44 in Haute-Loire, 48 in Puy-de-Dôme. Altitude, hence, appears to have an important effect.*

From the observation of 29 cases of vessels struck by lightning, at different times of the year, M. Arago concluded that, "at sea, lightning occurring in warm weather is much less dangerous than in cold."

In examining 103 instances of persons struck by lightning in France, we have found the following distribution: January none, February none, March 4, April 6, May 8, June 22, July 13, August 19, September 14, October 15, November none, December none. It follows, that, at least in France, the four cold months of the year may be said to be exempt from deaths by lightning.

As regards the sexes, 100 persons killed by lightning give us 67 men, 23 persons of whom the sex was not mentioned, and only 10 women. In Sweden, we find 5 men killed to 3 women; in England, 32 men and 11 women.

The maximum of persons killed by a single stroke of lightning, in the authorities we have been able to consult, has not exceeded the number of 8 or 9.

Animals are frequently more injured than the human species. Frequently entire flocks have been destroyed by one stroke of lightning. According to M. Abbadie, a single stroke killed two thousand sheep in Ethiopia.

In a number of instances, the shepherd, the horseman and the sportsman have been spared, whilst the thunderbolt struck cattle, horses and dogs.

Of 107 individuals killed by lightning from 1841 to 1854, we find 21 mentioned as having perished *under trees*.

We should add, however, that the place of death is not always mentioned, whence we have a right to infer that of the 1308 persons killed *instantaneously* in France from 1835 to 1852, three hundred at the least, would have escaped death if they had not sought shelter under trees.

Such facts deserve, we think, to be made public.

Fires caused by lightning are very numerous; their number has been as high as eight in a single week, in the departments of Meuse, of Moselle, of Menthe and Vosges.

* . . . . Feriuntque summos,

Fulmina montes. (Horat. i. 11, od. 7, ad Lic.)

The little kingdom of Wurtemberg, alone, shows, from 1841 to 1850, one hundred and seventeen fires caused by lightning.

Immense losses result by it to the sea service. From 1829 to 1830, during a period of fifteen months, five English vessels of war were struck by lightning. The Resistance and the Lynx were completely annihilated by it. The official reports to the English government show that the damage formerly caused by lightning to the royal service was never less than from 6,000 to 10,000 pounds sterling, annually, (150,000 to 250,000 fr.) In 200 cases of thunder-stroke, 300 sailors were killed or wounded; 100 main-masts, worth from 1000 to 1200 pounds each (25,000 to 30,000 fr.) were entirely destroyed. In the period alone, from 1810 to 1815, 35 vessels of the line and 35 frigates, besides other vessels of less importance, were put out of service in consequence of being struck by lightning. Since all the vessels of the royal marine, however, have been provided with lightning rods, the official reports have not mentioned the occurrence of any damage from this cause.*

It has been often stated that lightning has never set fire to powder contained in magazines. We have but one objection to make. It set fire to the powder magazine of Tangier on the 4th of May, 1788, to the magazine of Luxembourg, on the 26th of June, 1807, and to the magazine of Venice, on the 9th of November, 1808. Finally, in 1769, in falling, it struck the powder magazine of Brescia, destroying the sixth part of the dwellings there, and causing the death of three thousand persons.

These facts are sufficient, we think, to show the extent of the evil, and the necessity of our serious attention to it.

In a second discourse, we shall resume the symptomatology of the accidents caused by lightning, as well as the present state of science of the pathological anatomy of these accidents.

There are two points, as important as they are curious, in the medical history of death by lightning, we would wish to call the attention of the Academy to. 1st. The images (perhaps photographic) made upon persons struck. 2d. Of death, the attitude remaining that in which the person was, when struck. These two facts have not only a scientific interest; they may yet acquire a high importance in *legal medicine.*

* Sir Snow Harris, Rudimentary Electric. London: 1851. P. 188

As far back as 1786, two members of the Ancient Academy of Sciences cited, according to Franklin, the history of a man who, whilst standing at the door of a house, saw the lightning strike a tree which was before him. He found, says the report, *the fac-simile of the tree* upon his breast.

The circumstance was attributed to chance, or what was thought more likely, to a casual sanguineous effusion.

An analogous fact, which we had occasion to observe ourselves, determined us to make some researches, and this is the result of our investigation.

A woman of Lagun was struck by lightning: a flower which was in the course of the electrical current, was sketched upon her leg, and she preserved the trace of it during the remainder of her life.

In the roadstead of Zante, a sailor after being struck by lightning, discovered upon his breast the number 44, identical with that fixed in metal to the rigging of the ship.

In 1825, lightning struck the brigantine Il Buon Servo, and on the back of a sailor killed by it, was found the image of a horse shoe of the dimension of the iron nailed to the mizzen-mast.

On the 9th of October, 1836, in a storm near Zante, the young Politi was struck by lightning and killed. Doctor Dicapulo, charged by the Coroner to proceed to the examination of the body, mentions in his report the following facts:

"Young Politi, lying upon a bed, was dressed in a cotton jacket of a dark color, linen pantaloons, and waistcoat spotted with flowers. He wore a cravat of black silk, and a white sock upon his left foot; his right foot being naked. His half-boot, lying at the foot of the bed was ripped, and all his clothes, partly torn, were found burnt upon his back."

"M. Dicapulo adds, that in the right pocket of his coat were found a snuff-box and handkerchief, and in the left a parcel containing cream of tartar, &c.

Having entirely undressed him, says he, we saw about his loins a tight linen belt, and in the lining of this belt we found fourteen pieces of gold enveloped in two packages of paper; one in the right side contained a Spanish pistole, three guineas and two half guineas; that which was on his left side enclosed another Spanish pistole, four guineas, half a guinea, and two Venice sequins. Neither of these pieces, nor the paper, nor the linen, presented the slightest mark of scorch or burn. "Under his right foot a wound or gash of more than an inch in length, made us imagine that the lightning had penetrated by this extremity, and the passage was traced the whole length of the body; the leg and the right thigh, the buttocks and the back up to the neck, were strongly marked of a blackish color, and in all these parts the skin presented slight rents or ramified lacerations; the hair upon his body was almost all burnt, as well as his eye-lashes, his eye-brows, and his hair, particularly that behind his head. Little brown spots of the form and size of a lentil were scattered over his face. Finally, what appeared to us as most extraordinary," says Dr. Dicapulo, "the body had in the middle of the right shoulder, six circles, which preserved their flesh color, and appeared much more decided from the blackish color of the skin. These circles, one after the other, touching each other in one point, were of different sizes, corresponding exactly with the pieces of gold which the young man had in the right side of his belt, all of which was certified to by the coroner and by all the witnesses after the comparison was made.

We came to the conclusion that the unfortunate Spiridione Politi was struck by an ascending stroke of lightning, which, entering his right foot, traversed his whole body, burning the hair upon the surface, and penetrating the flesh, all of which was proved by the scarifications, the spots and the bronzed color of the skin; that the bituminous smell was owing to the decomposition of the fat of the body and the burning of his clothes; that the pieces of gold strongly attracted the electrical current, increased it, and carried the impressions to the extremity of the conductor, where they were fixed when it passed through the space which separated it from the window, by which it escaped.

I should not have been astonished if the electricity had melted or united the pieces of gold which were in its passage, leaving untouched those which were in the paper on his left side; but in granting, not without some difficulty, that the electric fluid may seize the impression of certain bodies which it meets with, and carry them to a distance, I do not understand how six pieces, piled up together, could be thus represented distinct and in a row. Many witnesses attest the fact; let us content ourselves with noting it, and collecting other facts of the same kind, inexplicable though they may appear to us."

In 1841, in the department of Inde et Loire, a magistrate and a miller boy, in the neighborhood of a poplar tree, were struck by lightning. Upon the breasts of both were found marks precisely similar to the leaves of the poplar.

Let us pass to the phenomena, which, for brevity, we shall call *death standing*.

We have seen the body of a man, who, after being killed and stript of clothing by lightning, remained in an upright position.

Cardan tells of eight harvestmen, or reapers, who took their repast under an oak; having been struck by lightning they retained their attitudes after death. One appeared to be still eating, another drinking, and another one extending his hand to his glass.

Father Beccaria and Dr. Gabrielli relate the history of men remaining in an upright position, who were killed by the electric fluid. We are familiar with several other facts of the same kind.

We all remember that in July, 1819, when the church of Chateau-neuf-les-Moustiers having been struck by lightning, *all* the dogs that were in the church were found dead, but on their legs.

M. Desormery has described a flash which struck him in 1849, and killed close by him a goat, which was found standing on its hind legs, still holding in its mouth a green branch.

Finally, among the most surprising facts of this kind, an instance is given of a priest who was struck by lightning while on horseback. The animal proceeded on his course and brought back the rider, dead it is true, but in the usual attitude of a man on horseback.

Plutarch speaks of a Spartan soldier, who, after vain efforts to keep the dead body of a man in an upright position, abandoned his design with these remarkable words, "Decidedly there was something within this." Well, what the Spartan soldier attempted in vain, is brought about by the action of lightning.

To sum up; 1st, the accidents caused by the electric fluid are serious and much more numerous than have hitherto been supposed.

2d. The mean annual number struck by lightning in France appears to exceed 200.

3d. The maximum of the accidents in France corresponds to the departments of the central table lands and mountainous districts.

4th. Women perish in a relatively small proportion.

5th. Among one hundred individuals thunder struck, twenty-five perish under trees.

6th. The upright death, or rather death with the attitude unchanged, and the photographic images, are phenomena of sufficient frequency for them to be taken into consideration in legal medicine.—*Gazette Medicale.*

The following newspaper extract was obligingly sent us by a friend:

"*An Electrotype.*—The Patterson (N. J.) Intelligencer gives a curious incident of the late thunder storm.

"A little girl was standing at a window before which was a young maple tree. After a brilliant flash of lightning, a complete image of the tree was found imprinted on her body. This is not the first instance of the kind, but it is a singular phenomenon."—*Public Ledger.* Ed.

